# Primary Cell Lines From Feathers and Blood of Free-Living Tawny Owls (Strix aluco): A New *In Vitro* Tool for Non-Lethal Toxicological Studies

**DOI:** 10.3389/fgene.2022.856766

**Published:** 2022-05-16

**Authors:** Ingvild Buran Kroglund, Sara Kristiane Kjærgård Eide, Jan Eivind Østnes, Rolf Terje Kroglund, Jan-Erik Frisli, Courtney Alice Waugh

**Affiliations:** ^1^ Faculty of Biosciences and Aquaculture, Nord University, Steinkjer, Norway; ^2^ BirdLife Norway, Trondheim, Norway

**Keywords:** non-lethal methods, toxicology, tawny owl, cell lines, *in vitro*, fibroblast cells, feather

## Abstract

The validation of the use of primary cell lines from non-lethal matrixes of feathers and blood of nestlings of a wild bird species, the tawny owl (Strix aluco) is described. Tawny Owl Feather Fibroblast (TOFF) cells and peripheral blood mononuclear cells (PBMCs) were isolated and cultured from the pulp of the secondary wing feathers and whole blood respectively from free-living tawny owl nestlings. Cell growth was registered up until 48 h for both the PBMC cells and the TOFFs. The validation of these primary cell lines in free-living birds has the potential to advance the assessment of immunotoxicological effects in wildlife *via* non-lethal manner. They provide a key tool with which to study cell toxicity and responses to environmental stressors on a cellular level in wild bird species of interest.

## Introduction

Measuring the toxicological impact of pollutants and multiple stressors on free-living wildlife *via* non-lethal methods is logistically challenging. Utilising *in vitro* tools such as primary and secondary cell lines can make it possible to study the cellular responses to stressors in wildlife *via* a non-destructive manner. For example, *in vitro* studies on blood primary cell lines of peripheral blood mononuclear cells (PBMCs) have shown to be good substitutes for exposure experiments in live animals since they represent an attractive tissue source in molecular and immunologic studies (Acosta Davila and Hernandez De Los Rios, 2019). They can serve as sentinel tissue for monitoring physiological responses due to environmental stressors. The PBMC cellular model includes T and B cells (∼80%), natural killer cells (∼10%) and monocytes (∼10%) (Autissier et al., 2010). There are also several studies that describes how secondary cell lines have been used to study immunological responses to viral infection and pollutant exposure for e.g. the effects of PFOS and PCBs on chicken fibroblasts (Waugh et al., 2018; Castaño-Ortiz et al., 2019; Badry et al., 2020a), and p,p-DDE exposure on immortalised humpback whale fibroblast cell lines isolated from the dermal connective tissue of skin biopsies (Burkard et al., 2015).

Primary cell lines, however, are a more biologically relevant option when compared to secondary (or immortalized) cell lines that have lost the true characteristics of the original tissue from which they are isolated. Serial passaging is known to cause genotypic and phenotypic variation in cell lines. Variation can often be so far from that of the original tissue to where they do not adequately mimic the *in vivo* environment. Primary cells can be of two types–adherent or suspension. Adherent cells require attachment for growth, usually derived from tissues of organs. Suspension cells do not require attachment for growth and are mostly isolated from the blood system. Although primary cells have a limited lifespan, they offer many advantages compared to secondary cell lines. When performing primary cell culture, there is the opportunity to study individuals and not just cells. Several factors such as age, sex, health status can then be considered when building an experimental model. Such individual variability and tissue complexity can only be achieved with the use of primary cells and are difficult to replicate with cell lines that are systematic and uniform in nature and do not capture the true diversity of a living tissue.

Validating the use of primary cell lines from wildlife to study multiple stressors in ecosystems is therefore warranted. Blood samples are the obvious first matrix choice. PBMCs are already routinely isolated and cultured *in vitro* for many validated downstream applications from personalised medicine to veterinary medicine, and wildlife research. However, taking blood samples from live animals can be logistically challenging and requires specialised expertise in the handling and extracting of blood. The amount of blood that can be taken from an individual to minimise adverse outcomes is also limited, especially in smaller species. Other non-destructive matrixes could then be explored for example skin biopsies from free-swimming humpback whales (Burkard et al., 2015), and wing punches from bats (Yohe et al., 2019) have both been used to produce cell lines.

For birds, where the focus of this study lies, cell lines have previously been isolated from the pulp of a feather, which is in the center of a developing feather consisting of living connective tissue. The pulp consists of fibroblasts and extracellular matrix including fibronectin and laminin (Davidson et al., 2021). Blood vessels and nerves enter the pulp *via* the dermal papilla in the feather follicle during the growing phase. Through these connections, nutrition can be provided to the growing feather follicles (Lin et al., 2013). Fibroblasts are known for repairing the extracellular matrix (ECM) during wound injuries (Davidson et al., 2021). These cells are also active in modulating immune responses in the stage of detecting pathogenic stimuli (Davidson et al., 2021). Fibroblasts can detect pathogen-associated molecular patterns, activate signalling pathways to recruited leukocytes (B- and T-cells) and then regulate their activity (Davidson et al., 2021). Since the fibroblast’s are so active during an immune response, they work as excellent cells in experiments while looking for immunological effects during pollutant or pathogen exposure. Feathers in the developing stage will have a greater amount of tissue compared to pulp from mature feathers (Xi et al., 2003). This means that the pulp present in the feather is affected by the phase of development of the feather. Teleoptile feathers, second generation feathers, is when the feathers are developing the most and are referred to as juvenile feathers (Yu et al., 2004).

According to the Norwegian Regulations cf. §7, plucked feathers are classified as a “light stressful attempt,” and are thereby less invasive than taking blood samples which is classified as a “moderate stressful attempt.” In addition, feathers are simple to collect, store, transport and use for analysis without causing damage to the birds, and they have many advantages that make them excellent non-destructive tool (Furness and Greenwood, 1993; García-Fernández et al., 2013; O'Sullivan and Sandau, 2014; Borghesi et al., 2016).

From a toxicological standpoint, wild birds, especially those high up the food chain, are useful organisms for monitoring long-term and large-scale changes of pollutants in the biological environment (Burger, 1993; Furness and Greenwood, 1993; O'Sullivan and Sandau, 2014). They are easy to observe, sensitive to environmental changes, and can accumulate large and harmful amounts of environmental pollutants (Seco Pon et al., 2011; García-Fernández et al., 2013; Espín et al., 2016). Birds are mainly exposed to pollutants through ingestion of contaminated food and water (Seco Pon et al., 2011). Many pollutants can subsequently bioaccumulate in different body tissues of birds such as blood, feathers, liver, kidney, brain, muscle, and bone (Jaspers et al., 2009; Jaspers et al., 2013; Espín et al., 2016; Løseth et al., 2019).

Biomonitoring of raptors is of particular interest. Since they often forage at the top of the food chain, they might be expected to bioaccumulate high levels of metals and other pollutants (Burger, 1993; Dauwe et al., 2003; Espin et al., 2014; Lohr, 2018; Hindmarch et al., 2019; Løseth et al., 2019). Historically, population declines were initially observed in species at the top of the food chain, and raptors can be vulnerable since bio-accumulative toxic substances tend to accumulate along their food chain. Tawny owls (Strix aluco) have recently been identified as one of the best Pan European biomonitoring bird species for monitoring the level of environmental pollution (e.g. toxic metals, anticoagulant rodenticides, pesticides and medicinal products) (Badry et al., 2020b). Tawny owls inhabit Western Palearctic with the vast majority of the breeding population in Europe (Holt, 2021). They are medium-sized, chiefly nocturnal owls (Cramp, 1985). All their body feathers are moulted once a year, while the wing feathers have a multi-annual moulting pattern (Jenni and Winkler, 2020). The population is large and the population trend appears stable (BirdLife International, 2022). According to IUCN Red list of threatened species tawny owls are listed as Least Concerned (LC) both in Europe and globally (IUCN red list, 2020). Tawny owls are resident species remaining within a restricted territory throughout the year, with hatching occurring in cultural landscapes. Their diet consists mainly of rodents and passerine birds (Cramp, 1985). Resident birds are completely dependent on the local environment for food, and can thus be used to monitor contamination in a more local terrestrial ecosystem (Burger, 1993; Peterson et al., 2019). Due to their abundancy and widespread distribution, their red list status (LC), their territoriality and residency, and the fact that non-lethal samples can easily be obtained from individuals in nest boxes, they are a key sentinel species for monitoring pollutants in terrestrial ecosystems across Europe (Debén et al., 2012; Bustnes et al., 2013; Bustnes et al., 2015; Eriksson et al., 2016; Seoane et al., 2018; Badry et al., 2020b).

Although raptors are highlighted as excellent biomonitoring species, there are currently no validated and standardised methods to study toxicological effects *via* non-lethal methods. Therefore, this study aimed to validate *in vitro* techniques for the initiation of cell lines from non-lethal matrixes of feathers and blood of nestlings of a wild bird species. The matrixes chosen represents one adherent primary cell line (i.e., TOFFs) and one non-adherent cell line (PBMCs). They were chosen because they are both: *1*) easily accessed without using lethal methods; *2*) represent cells with immunological importance (which will be the focus of our downstream applications). Overall, a standardised tool to assess immunotoxicology in free-living birds is lacking, yet very timely given the increase in epidemics such as avian influenzas (Adlhoch et al., 2021).

## Materials and Methods

### Sample Collection

This study was carried out in the northeast region of Trondheimsfjorden (64°N, 11°E) in central Norway. A nest box with three tawny owl nestlings was visited on the 26th of May 2021, 3 weeks after hatching. Blood samples (approx. 1–1.5 ml) were taken from the wing vein from each of the three nestlings using a pre-heparinized sterile 2 ml BD PlastiPak syringe with a 0.6 × 25 mm BD Microlance 3 23G no.16 and transferred to a heparinized tube. One secondary wing feather was taken from each of the nestlings and stored in zip bags. Samples were transported to the laboratory facilities within 1 h of collection. Samples were collected as part of a larger ongoing project (animal ethics approval numbers from the Norwegian Food Safety Authority, FOTS ID 23120).

### TOFF (Tawny Owl Feather Fibroblasts) Cell Lines From Secondary Wing Feathers

**FIGURE 1:**
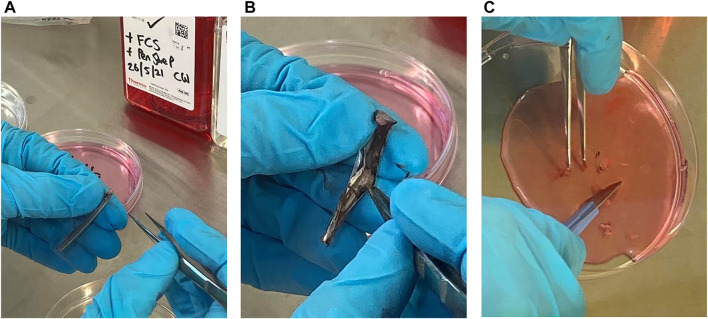
The process of separating cells from the calamus of feathers from Tawny Owls. **(A)** The calamus after being separated from the rest of the feather. **(B)** The calamus is cut open and the pulp is visible. **(C)** The pulp is separated from the rest of the calamus and diced into smaller pieces in cell culture media.

All the following steps were completed within 24 h of feather sampling. Feather samples from each individual was stored in separate plastic bags in the cooler or fridge (4°C) from sampling until the culturing was performed. The calamus was separated from the rest of the wing feather (Figure 1A) using a pair of sterile dissection scissors and swabbed with 70% ethanol for approximately 2–3 s in a petri-dish using sterile tweezers. Further, the calamus was rinsed with Dulbecco’s phosphate-buffered-saline with 2% foetal bovine serum (DPBS, Stemcell Technologies) in a petri-dish for 2–3 s. Then the calamus was rinsed with Dulbecco’s modified Eagle’s medium (DMEM, Thermo Scientific) added 5% foetal calf serum (FCS, Sigma, Oslo, Norway) and 1% of Pen-Strep, 100 U/ml Penicillin and 100 μg/ml Streptomycin (cell culture media) in a petri-dish for 2–3 s. At each step, a new sterile petri-dish was used. The calamus was then cut open vertically using a sterile scalpel and/or dissection scissors (Figure 1B). The pulp (cells) was separated from the calamus using sterile scalpel and tweezers in the petri-dish with cell culture media and after separation the pulp was placed in a new petri dish with new cell culture media. The pulp was diced into smaller pieces with the scalpel (Figure 1C) and the content with the cells were further transferred to a T-25 cell flask and added 10 ml cell culture media. Cells were then plated out into 96 well plates to allow for viewing under the microscope. Finally, the cells were incubated in a CO_2_ incubator at 38°C and 5% CO_2_ gas for 30 days.

### PBMC Cell Lines of Blood Samples From the Tawny Owl Nestlings

All the following steps were performed with blood samples stored within 24 h after blood sampling. The blood samples were stored in a dark area at room temperature. Approximately 2 ml of whole blood was transferred into a 15 ml sterile tube. The same amount of Dulbecco’s phosphate-buffered-saline with 2% foetal bovine serum (DPBS, Stemcell Technologies) was also transferred to the tube. The blood/DPBS mixture was then added to a separation tube (SepMate™ -15 (IVD), Stemcell Technologies) that contained 4.5 ml density gradient media (Lymphoprep™, Stemcell Technologies) and 80 µl Lymphocyte Enrichment Cocktail (RosetteSep™, Stemcell Technologies) to enrich lymphocytes from the whole blood samples. The separation tube was then centrifuged at 1200 *g* for 10 min for samples that were treated within 12 h, or 20 min for samples that were treated within 12–24 h, with the break on. The supernatant with the PBMCs containing the white blood cells (WBCs) was poured off into a new 15 ml tube. The WBC suspension was then washed with the same amount of DPBS with 2% FBS and centrifuged at 300 *g* for 10 min. The supernatant was discarded, and the pellet was resuspended. The cells resuspension was added into 10 ml Dulbecco’s modified Eagle’s medium (DMEM, Thermo Scientific) added 5% foetal calf serum (FCS, Sigma, Oslo, Norway) and 1% of Pen-Strep, 100 U/ml Penicillin and 100 μg/ml Streptomycin (cell culture media) in T-25 flasks. A subset of cells was then plated out into 96 well plates to allow for viewing under the microscope. Cells were incubated in a CO_2_ incubator at 38°C and 5% CO_2_ gas for 30 days.

### Cell Growth and Maintenance

Cells in the 96 well plates were checked daily and photographed weekly. Cell culture media was added to the cell cultures approximately once a week. Photos were taken either with an apple iPhone through the lens of a Leica DM1000/DM3000 microscope or on the EVOS XL Core image software.

## Results

**FIGURE 2:**
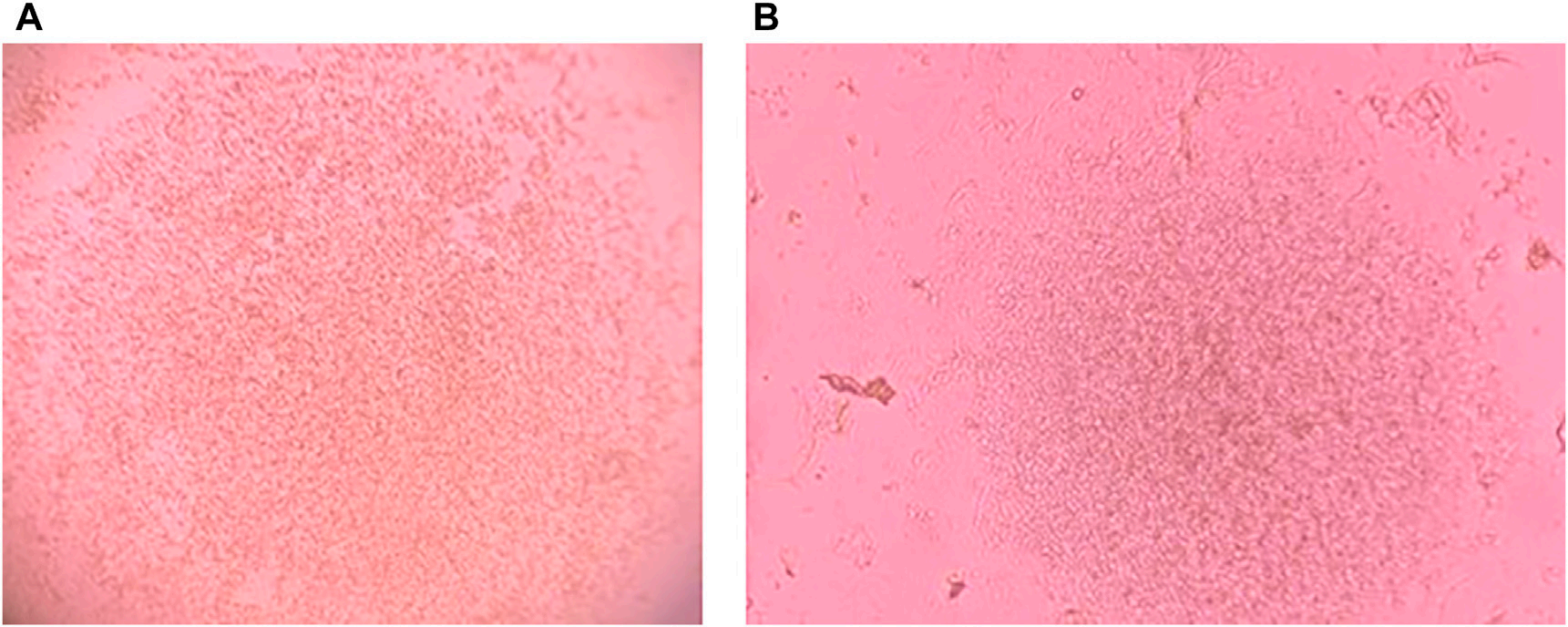
Cell growth after 5 days of incubation at 38°C and 5% CO_2_ of **(A)** Peripheral Blood Mononuclear Cells (PBMCs) isolated from the whole blood of free-living Tawny Owl nestlings and **(B)** Tawny Owl Feather Fibroblasts (TOFFs) isolated from the secondary wing feather of free-living tawny owl nestlings. Photo taken with iPhone 11 PRO down the lens of Leica DM1000 microscope.

**FIGURE 3:**
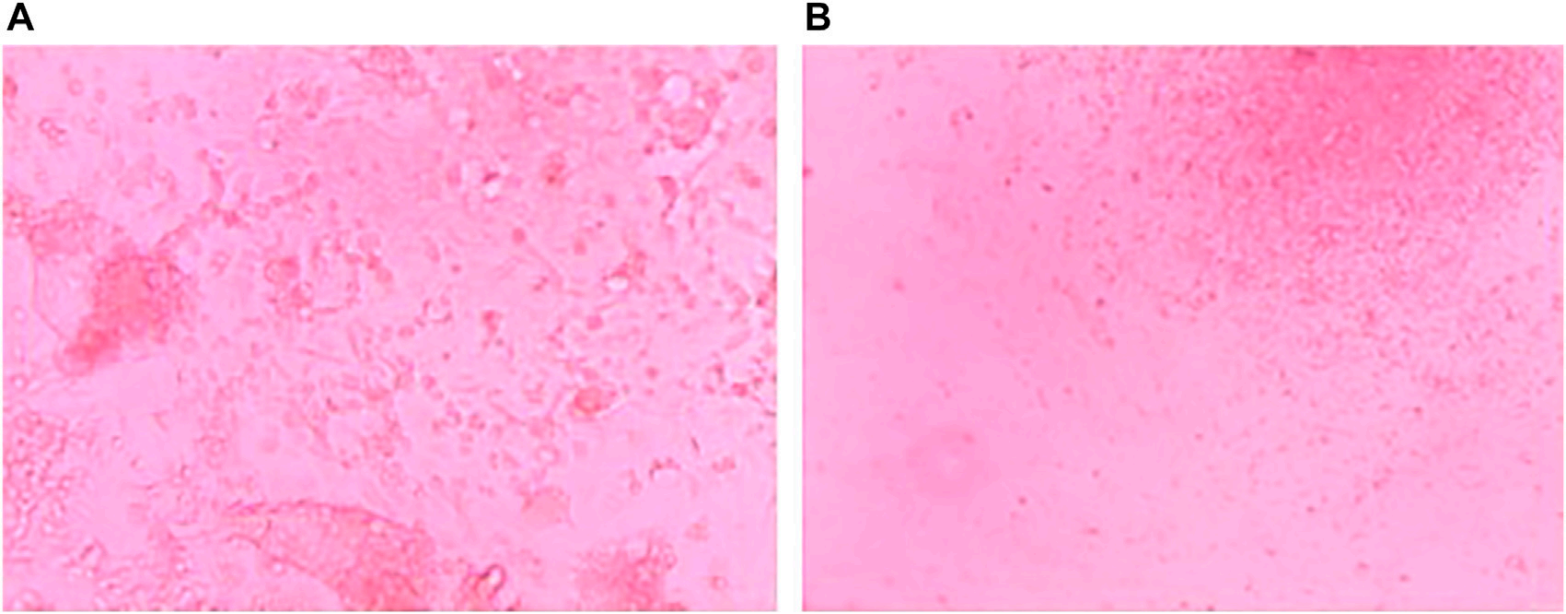
Cell growth after 30 days of incubation at 38°C and 5% CO_2_ of **(A)** Peripheral Blood Mononuclear Cells (PBMCs) isolated from the whole blood of free-living Tawny Owl nestlings and **(B)** Tawny Owl Feather Fibroblasts (TOFFs) isolated from the secondary wing feather of free-living Tawny Owl nestlings. Photo taken with a Leica camera attached to a DM3000 microscope.

Growth of PBMCs and TOFFs were registered in the 96 well plates after 5 days (Figures 2A,B, respectively). The PBMCs and TOFFs continued to proliferate in the 96 well plates until day 30 when the experiment was terminated (Figures 3A,B, respectively). At each time point (once a week) some cells (100 µl) were transferred to a microscope slide and stained with (100 µl) trypan blue to determine viability.

## Discussion

Non-lethal methods for studying the immunological health from a toxicological perspective of wildlife species are lacking. Here we have validated the use of primary cell lines from feathers (fibroblasts) and blood (PBMCs) from a free-living raptor species, the tawny owl for this purpose. Fibroblasts and PBMCs have key immunological properties (Waugh et al., 2018; Acosta Davila and Hernandez De Los Rios, 2019), therefore, there are many future implications for the advancement of assessing the immunotoxicological effects in wildlife in non-lethal manner.

Historically, cell lines have been based on domestic species like the domestic chicken (Gallus gallus domesticus) or quail (Coturnix coturnix) (Xi et al., 2003; Cardoso et al., 2020). By validating the use of primary cultures from blood and feather cells, *in vitro* studies can be further expanded into wildlife, instead of model species. This non-lethal method of a primary cultures creates possibilities of expanding the number of species and individuals to look for pollutant effects and investigate biological processes rather than the sacrificing of one individual to immortalize a secondary cell line; which are not as biological relevant as primary cell lines. Which in turn, create possibilities of making an environment of cell exposure for specific species that are suffering from pollution exposure in the wild. By using cell lines from wildlife species of interest, the results have the potential to have less inter-species bias (both between different wild bird species and between domesticated vs wild bird species).

The next step in this procedure will be to utilise Tawny Owl PBMCs and TOFFs for immunotoxicology studies by exposing the cells to environmentally relevant levels of pollutants (e.g., in our case, to study the immune responses to heavy metals, PFASs and rodenticides on a cellular level). PBMCs can be used for classic lymphocyte proliferation assays, which makes it feasible to measure toxicity of the cells. The assay discovers the compound’s ability to block or cause biological activity without having toxic effects on cells (Creative Bioarray, 2022), which makes it possible to investigate the pollutant levels in the cells before further *in vitro* experiments. TOFF cells can be used for infection/exposure studies with pollutants and pathogens of choice (Waugh et al., 2018; Castaño-Ortiz et al., 2019; Badry et al., 2020a)). Previous studies performed on domestic chicken secondary cell lines have already demonstrated the beneficial use of cell lines in exposure studies where cell cultures have been exposed to environmentally relevant concentrations of pollutants such as PFOS and PCBs, and infected with DNA- and RNA viruses to investigate the downstream immunological responses (e.g. the expression pattern for mir-155, pro-inflammatory TNFα and IL-8, transcription factor NF-κB1, and anti-inflammatory IL-4) on a cellular level (Waugh et al., 2018; Castaño-Ortiz et al., 2019; Badry et al., 2020a). These studies all showed concerning modulations in the immunological responses after exposure to different pollutants (PFOS and PCB). Validating the use of primary cell lines (TOFFs and PBMCs) is an integral step towards performing more biologically relevant *in vitro* experiments (e.g., pollutant exposure and/or pathogen infections to investigate the toxicological and immunological responses) on a cellular level of any wild bird species of interest. For further studies, it is also possible to increase the levels of pollutants in the *in-vitro* studies compared to the current environmental concentrations to simulate how future contamination levels will affect the immune response.

Current anthropogenic threats makes raptors more vulnerable to toxicants as they are susceptible to bioaccumulate high levels of metals and other pollutants (Burger, 1993; Dauwe et al., 2003). Further, their prey, which includes migratory passerines, could also transfer infections such as avian influenzas. Indeed, raptors have been recently experiencing increasing incidents of high pathogenic avian influenza virus (HPAIV) infections associated with neurological diseases, necrosis in essential organs (heart, pancreas, lung and brain), and death around the globe (Krone et al., 2018; Shearn-Bochsler et al., 2019; OIE, 2021; NVI, 2022). Since top predators might experience harmful concentrations of pollutants as well as pathogen infections, it is especially important to focus on how they will respond to the multiple stress of a new infection when already immunocompromised *via* pollution levels. However, care must be taken when interpreting the results on cell lines because they cannot completely replace or reflect the whole complexity of the physiological processes that takes place in an entire organism (Kaur and Dufour, 2012). However, they can still provide helpful information about important biological processes and responses, especially for wildlife species where it is often considered unethical and counterintuitive to the aims of saving a species if it is used for lethal toxicology studies.

## Data Availability

The original contributions presented in the study are included in the article/Supplementary Material, further inquiries can be directed to the corresponding author.
